# Predictive value of TCM clinical index for diabetic peripheral neuropathy among the type 2 diabetes mellitus population: A new observation and insight

**DOI:** 10.1016/j.heliyon.2023.e17339

**Published:** 2023-06-16

**Authors:** Zhikui Tian, Yadong Fan, Xuan Sun, Dongjun Wang, Yuanyuan Guan, Ying Zhang, Zhaohui Zhang, Jing Guo, Huaien Bu, Zhongming Wu, Hongwu Wang

**Affiliations:** a School of Health Sciences and Engineering, Tianjin University of Traditional Chinese Medicine, Tianjin, 301617, China; b Nanjing University of Chinese Medicine, Nanjing, 210023, China; c Affiliated Hospital of Nanjing University of Chinese Medicine, Nanjing, 210004, China; d College of Traditional Chinese Medicine, North China University of Science and Technology, Tangshan, 063000, China; e Fengnan District Hospital of Traditional Chinese Medicine, Tangshan, 063000, China; f Surgery of TCM, Second Affiliated Hospital of Tianjin University of TCM, Tianjin, 301617, China; g Department of Endocrinology, Shandong Provincial Hospital Affiliated to Shandong First Medical University, Jinan, Shandong, 250021, China

**Keywords:** Diabetic peripheral neuropathy_1_, Type 2 diabetes mellitus_2_, Risk factor_3_, Symptoms of TCM_4_, Tongue features_5_

## Abstract

**Aims:**

The objectives of this study were to identify clinical predictors of the Traditional Chinese medicine (TCM) clinical index for diabetic peripheral neuropathy (DPN) in type 2 diabetes mellitus (T2DM) patients, develop a clinical prediction model, and construct a nomogram.

**Methods:**

We collected the TCM clinical index from 3590 T2DM recruited at the Second Affiliated Hospital of Tianjin University of Traditional Chinese Medicine from January 2019 to October 2020. The participants were randomly assigned to either the training group (n = 3297) or the validation group (n = 1426). TCM symptoms and tongue characteristics were used to assess the risk of developing DPN in T2DM patients. Through 5-fold cross-validation in the training group, the least absolute shrinkage and selection operator (LASSO) regression analysis method was used to optimize variable selection. In addition, using multifactor logistic regression analysis, a predictive model and nomogram were developed.

**Results:**

A total of eight independent predictors were found to be associated with the DPN in multivariate logistic regression analyses: advanced age of grading (odds ratio/OR 1.575), smoke (OR 2.815), insomnia (OR 0.557), sweating (OR 0.535), loose teeth (OR 1.713), dry skin (OR 1.831), purple tongue (OR 2.278). And dark red tongue (OR 0.139). The model was constructed using these eight predictor's medium discriminative capabilities. The area under the curve (AUC) of the training set is 0.727, and the AUC of the validation set is 0.744 on the ROC curve. The calibration plot revealed that the model's goodness-of-fit is satisfactory.

**Conclusions:**

We established a TCM prediction model for DPN in patients with T2DM based on the TCM clinical index.

## 1 Introduction

Diabetes mellitus has been diagnosed in nearly 537 million people worldwide as a result of changes in dietary structure, residents' lifestyles, and economic development. The incidence rate is expected to rise to 11.3% in 2030 and 12.2% in 2045 [1]. From 2013 to 2018, the prevalence of diabetes mellitus in China rose from 10.9% to 12.4% [2]. In 2021, China has the highest percentage of both diagnosed and undiagnosed cases staggering 140.9 and 72.8 million people, respectively.

Although the awareness and treatment rates of diabetes mellitus patients in China have increased in recent years, they are still not optimal. Lifestyle modification is the primary preventive measure for diabetes and its complications [3]. Diabetic peripheral neuropathy (DPN) is a prevalent and severe chronic complication of diabetes mellitus that affects approximately 30% of patients. It is linked to lower extremity amputations, painful neuropathy symptoms, and an increased risk of death [4]. Diabetes-related foot ulcers are characterized by foot ulceration or deep tissue destruction caused by DPN [5], which affects about 20 million people worldwide each year [6]. Obesity, hyperglycemia, dyslipidemia, impaired neurotrophic support and insulin signaling, and microvascular diseases all cause downstream oxidative stress, mitochondrial dysfunction, and inflammation, ultimately leading to cellular dysfunction and death [[7], [8], [9], [10], [11]]. The management of diabetic peripheral neuropathy encompasses targeted treatments for neurological symptoms and strategies to prevent DPN's progression and address foot complications [4]. However, the onset of DPN is insidious, and up to fifty percent of patients have no typical conscious symptoms, necessitating that clinicians and patients pay greater attention to early screening for DPN to provide timely and effective diagnosis and treatment [12].

Nerve conduction studies remain the gold standard for diagnosing and assessing the severity of DPN [13,14]. Before attributing neuropathy to diabetes mellitus, a thorough clinical history and physical examination should be conducted to rule out neuropathy caused by a neurological or orthopedic disease. After the exclusion of lesions, five screening tests (ankle reflex, vibration sensation, pressure sensation, pinprick sensation, and temperature sensation) should be performed for clinical symptoms of neuropathy in diabetes mellitus (including pre-diabetes mellitus), such as pain, numbness, and paresthesia [4,15,16]. Generally speaking, these DPN diagnostic strategies are adequate in clinical settings. Neurology consultations and neurophysiological testing are advised when conditions such as atypical symptoms, signs, or diagnostic uncertainty arise [13]. Academics have investigated and validated early screening, diagnostic methods, and devices for DPN. Several innovative point-of-care devices have the potential to facilitate the early diagnosis of DPN in potentially more treatable conditions [17]. Still, their accuracy and cost-effectiveness must be studied further [18]. Scored clinical assessments provide a standardized, objective, quantitative, and reproducible method for diagnosis, diagnosing, and grading the severity of DPN [15]. However, these methods are time-consuming and challenging to implement in clinical settings. Given that DPN is a relatively complex pathological process, it has become imperative to develop a simple, distinct, noninvasive prediction method for early clinical diagnosis with multiple influencing factors to estimate the risk of DPN or the current condition in a busy medical setting.

The primary objective of this research is to develop a scientific prediction model for the risk of DPN occurrence in diabetes mellitus to address the critical need for early screening. TCM diagnosis is based on four processes: sight, hearing, odor, and questioning. The most common method is to check the tongue and pulse. According to research, tongue imaging is linked to type 2 diabetes mellitus (T2DM) and can be used to screen for T2DM [[61], [62], [63], [64],^68^]. To our knowledge, no studies have been done to create a clinical prediction model for DPN related to TCM that combines multiple indicators, TCM symptoms, and tongue characteristics.

In this study, a total of 4723 cases of T2DM were included, the potential relationship between DPN and TCM indicators was explored, and a nomogram model was developed to facilitate the early diagnosis of DPN by TCM clinical practitioners.

## 2 Materials and methods

This study was approved by the institutional ethics committee of Tianjin University of Traditional Chinese Medicine (approval number: TJUTCM-EC20190004), and all participants had to provide informed consent before the study's initiation.

### 2.1 Study design and participants

Patients with T2DM admitted between January 2019 and October 2020 to the endocrinology department and traditional Chinese medicine surgery department of the Second Affiliated Hospital of Tianjin University of Traditional Chinese Medicine, Tianjin, China, were included in the cross-sectional study. The diagnosis of T2DM was based on a previous diagnosis of fasting plasma glucose (FPG) ≥ 7.0 mmol/L or 2 h plasma glucose (PBG) ≥ 11.1 mmol/L at the Second Affiliated Hospital or confirmed diagnosis of DPN based on a positive 128 Hz tuning fork and 10 g monofilaments [19]. The exclusion criteria were as follows [1]: age below 18 [2]; failure to complete the questionnaire [3]; pregnant or lactating women [4]; unable to cooperate with the complete tongue image collector [5]; incomplete clinical data. Based on the criteria above, a total of 4723 patients were enrolled in our study. The dataset was randomly divided into two groups: 70% (n = 3297) comprised the training group, and 30% (n = 1426) formed the validation group. The training group was used to construct the model, while the validation group was used to evaluate the model's capabilities.

### 2.2 Baseline data collection and processing

Each participant's age and gender were adequately recorded. During the study, patients who smoked at least one cigarette per day were considered smokers. The BMI was calculated by dividing weight in kilograms by height in meters squared. The Chinese guidelines for the prevention and control of overweight and obesity in adults classify BMI into: <18.5, 18.5–23.9, 24–28, and >28 kg/m^2^ [20].

### 2.3 Clinical symptoms of TCM collection

Using a standardized questionnaire (Information Record Form of TCM Clinical Four Diagnostics) developed by Shanghai University of Traditional Chinese Medicine, trained researchers from the School of Health Sciences and Engineering at Tianjin University of Traditional Chinese Medicine interviewed each patient and collected clinical symptom information. “Yes” responses were given 1 point, and “No” responses, 0 [21].

### 2.4 Collection of tongue features

Two researchers identified tongue characteristics and recorded the findings in the questionnaires. The Tongue Diagnosis Analysis-1 (TFDA-1) instrument developed by the national key research and development plan was used to collect tongue images [22]. Two experienced TCM experts were asked to review the images simultaneously; in the event of a disagreement, a third expert would decide the outcome.

### 2.5 Statistical analysis

The statistical analysis was performed using SPSS (26.0) and STATA (version 15.0). As appropriate, Chi-squared tests were used to compare the training and validation groups. All reported levels of statistical significance were two-sided, with a significance level of 0.05. Different variables were evaluated, and the odds ratio and 95% confidence interval (CI) were calculated using the logistic regression analysis method. Next, variables with a P value of less than 0.05 were added to the model. Statistical significant variables were included in the multivariate logistic regression analysis (backward: conditional). This study used the minimum absolute contraction and lasso regression to select the most valuable predictor candidates [23]. The LASSO regression analysis performed a 5-fold cross-validation for centralization and normalization of the included variables before choosing the optimal lambda value [24]. Finally, a nomogram model was developed to identify T2DM patients at risk for DPN.

### 2.6 Model evaluation

**Fig. 1:**
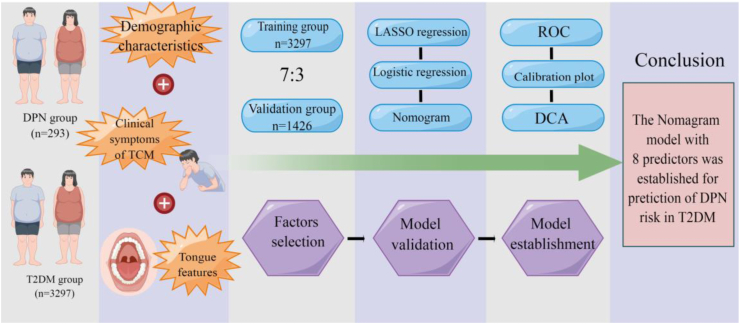
The flowchart of the TCM prediction model for DPN in T2DM patients based on the TCM clinical index.

The prediction model's efficacy evaluation focuses primarily on discrimination ability (discrimination), accuracy (calibration curve), and clinical applicability. The closer the AUC value is to 1, the better the prediction model is [25]. The calibration degree is an essential metric for assessing the precision of a disease risk model. Finally, a decision curve analysis was performed to evaluate the clinical efficacy of the model [26]. Fig. 1 depicts a Figdraw (www.figdraw.com) flowchart of this study. LASSO regression, multivariate logistic analysis, and the establishment of a nomogram model were performed using STATA (version 15.0).

## 3 Results

### 3.1 Patient characteristics

We analyzed three clinical variables: demographic characteristics, symptoms and signs of TCM, and tongue features collected via a standardized questionnaire with categorical variables between 2019 and 2021 [21]. The study included 4430 T2DM patients and 293 DPN patients. Finally, 4723 patients were divided into the training group (3297 individuals) and the validation group (1426 individuals) in this study. Based on the standard of ten events per candidate predictor parameter, this study's 25 predictors required at least 250 individuals with their respective events [27]. In addition, considering other sample size requirements for developing a clinical prediction model, the training group sample size of 3297 patients met the standard optimal sample size for further statistical analyses. This study included 1541 males and 1756 females, the majority aged 60–89 (61%). 45.7% of the patients were overweight (Table 1). The DPN group had more frequencies of age >60 years old than the T2DM group. The most frequent BMI interval in DPN patients was 24.6–28 (42.3%). The DPN group has more male members (57.3%) than female members (42.7%). Significantly more individuals in the DPN group (44.4%) were smokers than in the T2DM group (21.9%), and the frequency of purple tongue and the yellow coating was significantly higher in the DPN group than in the T2DM group (S1). Fig. 6 displays the tongue characteristics, the left column is the color of the tongue (Fig. 6a), the middle column is the tongue shape (Fig. 6b), and the right column is the color and shape of the coating (Fig. 6c).

**Table 1:** Clinical characteristics of the study participants.

Indicators	Clinical characteristics N (%)	Training group (3297)	Validation group (1426)	*P* value
Baseline Data	Age			0.533
	18～44	182 (5.5)	83 (5.8)	
	45～59	947 (28.7)	399 (28)	
	60～89	2010 (61)	862 (60.4)	
	>90	158 (4.8)	82 (5.8)	
	BMI			0.212
	<18.5	33 (1)	12 (0.8)	
	18.5～23.9	1307 (39.7)	609 (42.7)	
	24～28	1505 (45.7)	609 (42.7)	
	>28	450 (13.7)	196 (13.7)	
	Sex			0.610
	male	1541 (46.7)	655 (45.9)	
	female	1756 (53.3)	771 (54.1)	
	Smoke	755 (22.9)	346 (24.3)	0.309
Symptoms of TCM	Fatigue	1925 (58.4)	838 (58.8)	0.808
	Irritable	1306 (39.6)	546 (38.3)	0.393
	Forgetfulness	143 (4.3)	47 (3.3)	0.095
	Insomnia	1514 (45.9)	655 (45.9)	0.994
	Sweating	878 (25.6)	366 (25.7)	0.490
	Loose teeth	867 (26.3)	368 (25.8)	0.725
	Dry skin	549 (16.7)	254 (17.8)	0.330
	Dry mouth	1345 (40.8)	595 (41.7)	0.551
	Polydipsia	529 (16)	226 (15.8)	0.866
	Thirst does not drink much	86 (2.6)	40 (2.8)	0.700
	Polyuria	136 (4.1)	46 (3.2)	0.141
	Frequency of urine	426 (16.9)	170 (11.9)	0.342
Tongue Features	Crimson tongue	517 (15.7)	238 (16.7)	0.385
	Purple tongue	253 (7.7)	120 (8.4)	0.386
	Dark red tongue	367 (11.1)	149 (10.4)	0.490
	Enlarged tongue	348 (10.6)	122 (8.6)	0.035
	Spotted tongue	205 (6.2)	101 (7.1)	0.268
	Teeth marked tongue	838 (25.4)	343 (24.1)	0.320
	Fissured tongue	1196 (36.3)	563 (39.5)	0.036
	Yellow coating	873 (26.5)	392 (27.5)	0.471
	Less coating	221 (6.7)	107 (7.5)	0.320
	Thick coating	778 (23.6)	317 (22.2)	0.307
	Greasy coating	832 (25.2)	368 (25.8)	0.679

Note: Values are shown with numbers and proportions. P < 0.05 means a significant difference. BMI, Body mass index, Underweight <18.5 kg/m2, normal BMI range 18.5–23.9 kg/m2, overweight 24.0 ≤ BMI <28.0 kg/m2; obesity >28.0 kg/m2.

**Fig. 2:**
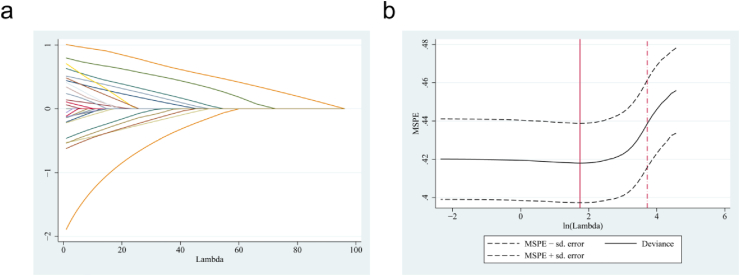
LASSO regression analysis for DPN in T2DM. Coefficient path diagram of 25 clinical features, (a) determined the optimal 8 clinical features with nonzero coefficients by deriving the optimal lambda. The MSPE versus lambda and dotted vertical lines were performed based on 5-fold cross-validation LASSO (b).

**Fig. 3:**
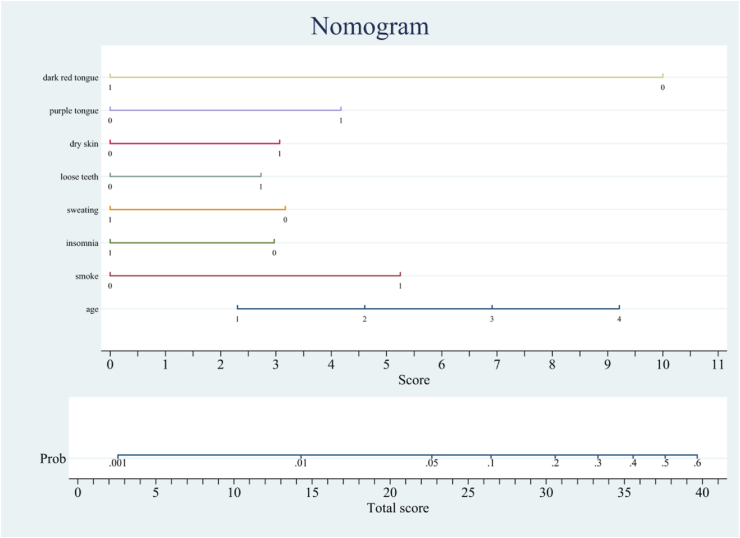
Nomogram prediction model of DPN. Risk factors of age, smoke, insomnia, sweating, loose teeth dry skin, purple tongue, dark red tongue for nomogram prediction model. It was established by the training group. A total point was calculated by combining appearance of smoke, loose teeth, dry skin, purple tongue and age grade. (For interpretation of the references to color in this figure legend, the reader is referred to the Web version of this article.)

**Fig. 4:**
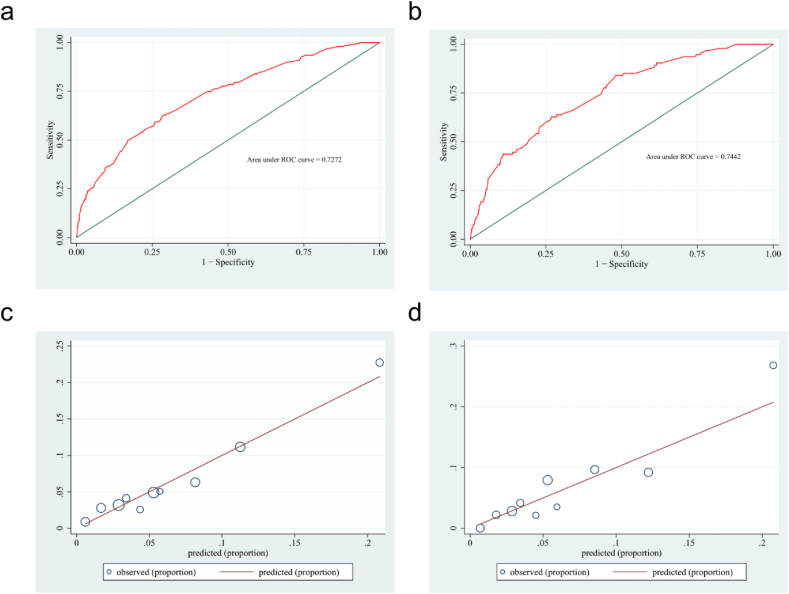
Receiver operating characteristic curve (ROC) validation of the DPN risk nomogram prediction. The x-axis represents the false positive rate of the prediction. The y-axis represents the true positive rate of the prediction, the red line represents the performance of the model of training set (a) and validation set (b). Calibration curves of the predictive DPN nomogram prediction model. The x-axis represents the predicted risk of DPN. The y-axis represents actual diagnosed cases of DPN, the red line represents a perfect prediction by an ideal model, the blue circle represents the performance of the training set (c) and validation set (d), the results illuminate the agreement between the predicted risks of DPN and the observed incidence of DPN. (For interpretation of the references to color in this figure legend, the reader is referred to the Web version of this article.)

**Fig. 5:**
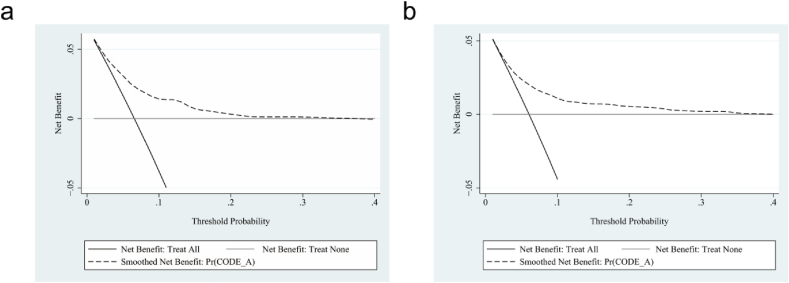
Decision curve analysis (DCA) was exhibited to estimate the clinical usefulness of DPN nomogram prediction model. The x-axis represents the threshold probabilities, the y-axis represents the standardized net benefit in DCA curves, and the black dotted line represents DPN nomogram, the solid black line represents the condition that all patients have DPN, and the gray line represents the condition that none have DPN. (a) From the training group, (b) from the validation group.

**Fig. 6:**
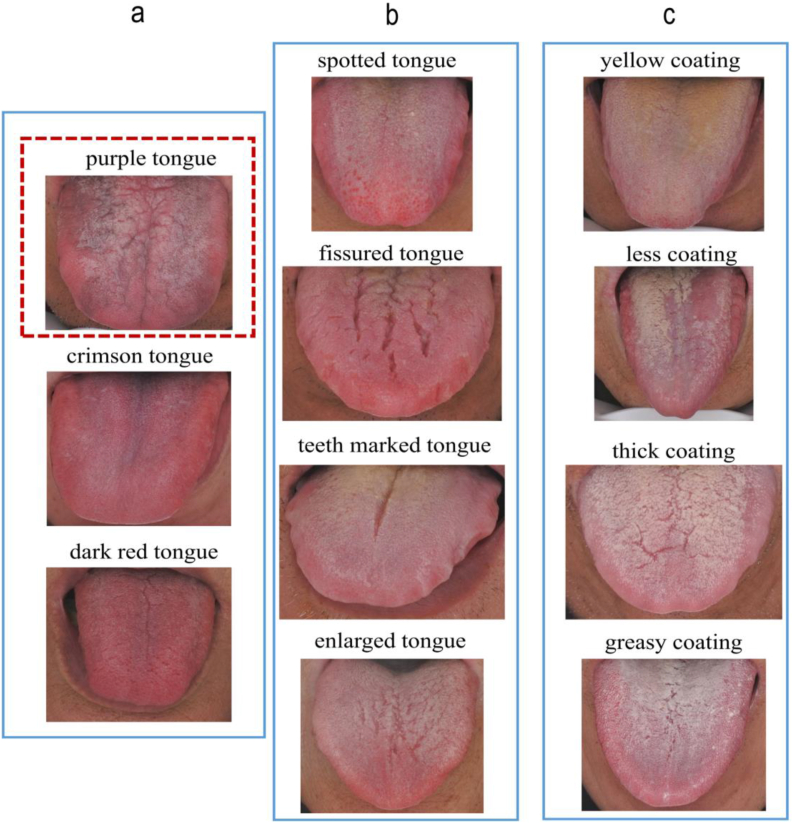
Tongue features Inside the red dotted box is the purple tongue, the left column is the color of the tongue, including Purple tongue, Crimson tongue, Dark red tongue (a); The middle column is the shape of the tongue, including Spotted tongue, Fissured tongue, Teeth marked tongue and Enlarged tongue (b); The column on the right is the color and shape of the coat, including Yellow coating, Less coating, Thick coating, Less coating (c). (For interpretation of the references to color in this figure legend, the reader is referred to the Web version of this article.)

### 3.2 Factor selection

In the training group, univariate analysis was used to identify risk factors associated with DPN in patients with T2DM. Significantly different predictors included age (OR 1.440, 95% CI 1.140–1.820, p = 0.002), sex (OR 0.558, 95% CI 0.416–10.747, p = 0.000), smoking (OR 2.763, 95% CI 2.061–3.705, p = 0.000), insomnia (OR 0.565, 95% CI 0.417–0.766, p = 0.000), sweating (OR 0.530, 95% CI 0.362–0.776, p = 0.001), loose teeth (OR 1.721, 95% CI 1.277–2.319, p = 0.000), dry skin (OR 1.958, 95% CI 1.412–2.716, p = 0.000), purple tongue (OR 2.709, 95% CI 1.827–4.017, p = 0.000), dark red tongue (OR 0.115, 95% CI 0.037–0.361, p = 0.000), yellow coating (OR 1.375, 95% CI 1.011–1.869, p = 0.042), and greasy coating (OR 0.639, 95% CI 0.441–0.925, p = 0.018). Other predictors were not significantly different (Table 2).

**Table 2:** Risk factors for DPN in T2DM patients according to the logistic regression model.

	Features	OR	Lower	Upper	*P* value
Baseline Data	Age	1.440	1.140	1.820	0.002
	BMI	1.221	0.999	1.493	0.052
	Sex	0.558	0.416	0.747	0.000
	Smoke	2.763	2.061	3.705	0.000
Symptoms of TCM	Fatigue	0.974	0.729	1.302	0.860
	Irritable	1.073	0.802	1.436	0.635
	Forgetfulness	0.794	0.366	1.721	0.559
	Insomnia	0.565	0.417	0.766	0.000
	Sweating	0.530	0.362	0.776	0.001
	Loose teeth	1.721	1.277	2.319	0.000
	Dry skin	1.958	1.412	2.716	0.000
	Dry mouth	0.974	0.727	1.305	0.860
	Polydipsia	1.342	0.937	1.924	0.109
	Thirst does not drink much	1.858	0.918	3.764	0.085
	Polyuria	1.539	0.836	2.834	0.166
	Frequency of urine	0.829	0.526	1.307	0.419
Tongue Features	Crimson tongue	1.202	0.827	1.746	0.335
	Purple tongue	2.709	1.827	4.017	0.000
	Dark red tongue	0.115	0.037	0.361	0.000
	Enlarged tongue	0.781	0.469	1.300	0.342
	Spotted tongue	0.966	0.529	1.763	0.910
	Teeth marked tongue	0.798	0.564	1.130	0.204
	Fissured tongue	0.844	0.622	1.145	0.275
	Yellow coating	1.375	1.011	1.869	0.042
	Less coating	1.325	0.790	2.222	0.286
	Thick coating	0.701	0.484	1.016	0.060
	Greasy coating	0.639	0.441	0.925	0.018

Through the shrinkage estimation method, the LASSO method can also select relevant features in high-dimensional data. In this study, the training group was subjected to LASSO regression. The modification of the penalty coefficient λ gradually reduced the number of model variables. A 5-fold cross-validation error was selected as the minimum λ+1 (lambda. 1se = 3.262) as the optimal value of the model (Fig. 2a–b), which included 8 of the 25 variables. Then, the eight variables obtained from the Lasso regression, including age, smoke, insomnia, sweating, loose teeth, dry skin, purple tongue, and dark red tongue were significantly different between the two groups. Similarly, 11 predictors were derived from univariate analysis, and a 5-fold cross-validation yielded the same result. In light of this, the logistic multiple regression model incorporated a model with eight independent predictors.

### 3.3 Model development

A logistic multiple regression analysis was conducted on the identified potential DPN predictors (p < 0.05). The predictors included in the prediction model were age (OR 1.575, 95% CI 1.236–2.006, p = 0.000), smoking (OR 2.815, 95% CI 2.079–3.811, p = 0.000), insomnia (OR 0.557, 95% CI 0.408–0.761, p = 0.000), sweating (OR 0.535, 95% CI 0.362–0.791, p = 0.002), loose teeth (OR 1.713, 95% CI 1.258–2.331, p = 0.001), dry skin (OR 1.831, 95% CI 1.303–2.574, p = 0.000), purple tongue (OR 2.278, 95% CI 1.514–3.428, p = 0.000), and dark red tongue (OR 0.139, 95% CI 0.044–0.441, p = 0.001) (Table 3). A DPN risk nomogram was also constructed based on our model (Fig. 3).

**Table 3:** Multivariate logistic analysis of risk factors to DPN.

Features	OR	Lower	Upper	*P* value
age	1.575	1.236	2.006	0.000
smoke	2.815	2.079	3.811	0.000
insomnia	0.557	0.408	0.761	0.000
sweating	0.535	0.362	0.791	0.002
loose teeth	1.713	1.258	2.331	0.001
dry skin	1.831	1.303	2.574	0.000
purple tongue	2.278	1.514	3.428	0.000
dark red tongue	0.139	0.044	0.441	0.001

### 3.4 Model validation

The ROC curve was used to evaluate the predictive model's discriminatory ability. During internal validation, the predictive nomogram in the training group demonstrated good discrimination (AUC = 0.727) (Fig. 4a). The curve indicated that the prediction nomogram had a good fit (AUC = 0.744) (Fig. 4b). In addition, the calibration curve showed that the predicted DPN incidence was consistent with the observed incidence in the training and validation groups (Fig. 4c–d).

### 3.5 Decision curve analysis

The decision curve analysis (DCA) was presented to assess our model's clinical utility. The DCA curves demonstrated that along with an increase in the probability threshold, referring to our predicted models when deciding whether to intervene can add net benefit compared to intervening in all patients or none (Fig. 5a–b).

## 4 Discussion

The prevalence of DPN among Chinese patients diagnosed with T2DM was greater than 50% [28,29]. DPN is characterized by a high incidence, an insidious onset, atypical clinical symptoms, and significant harm, all of which harm patients' quality of life. Since DPN is difficult to diagnose due to its unclear pathogenesis, early risk assessment, and prevention research is critical.

. In this study, T2DM patients were examined to determine the risk factors for DPN lesions and to develop a DPN nomogram risk prediction model that included eight variables: age, smoking, insomnia, sweating, loose teeth, dry skin, purple tongue, and dark red tongue. In addition, the ROC curve and DCA demonstrated that the model possesses excellent discriminatory and predictive abilities. This nomogram is a noninvasive, easy-to-use, and effective tool that could be used in the outpatient setting to perform a rapid assessment of DPN risk in patients diagnosed with T2DM, particularly in TCM clinics. It is a cross-sectional study of T2DM patients in the Tianjin region in which the patients' characteristics (demographic characteristics, tongue features, and symptoms) were systematically recorded and collected. Using the 5-fold cross-validation LASSO regression method, the 27 variables were initially reduced to 8 potential predictors. Then, we obtained 11 predictors from univariate analysis, ran 5-fold cross-validation LASSO regression, and obtained the same result.

According to previous research, the primary risk factors for DPN are diabetes duration, hyperglycemia, age, pre-diabetes, hypertension, hyperlipidemia, and smoking [30]. The 2020 Guidelines for the Prevention and Treatment of T2DM in China also note that patients with diabetes older than ten years are more likely to develop obvious DPN. A cross-sectional multicenter study in India revealed that the severity of DPN was significantly related to age and duration of diabetes [31]; the risk of DPN increased by 7% for each additional year of diabetes duration [32]. Age and abnormal glucose and lipid metabolism harm the vascular endothelium, disrupt the homeostasis of oxidative stress in the body, affect nutrient supply, and damage nerve cells [33]. A 9-year follow-up study involving 512,891 urban and rural adults in China revealed that long-term smokers had a 15–30% greater risk of developing diabetes than nonsmokers. Among urban men, the hazard ratio was greater the younger they started smoking and the more they smoked [34]. A meta-analysis of 84 prospective studies, including nearly 300,000 diabetes cases, found that active smoking was associated with an overall hazard ratio for reported diabetes of 1.37 (95% prediction interval 1.11–1.50) [35]. DPN is more prevalent in diabetic patients who smoke, are older than 40, and have inadequate blood glucose control. A recent study revealed that smoking is an independent risk factor for DPN and that the risk of DPN increases with the duration of smoking and the level of addiction. Notably, heavy smokers had poorer nerve conduction, which may be due to smoking-induced inflammatory cytokine release and impaired endothelial cell function [9]. Smoking is also significantly associated with the risk of diabetic foot, as the daily tissue hypoxia caused by smoking can result in vascular and neuropathy in diabetic patients' extremities [36].

Patients with DPN can effectively avoid infection and reduce the incidence of diabetic foot by protecting their skin. However, DPN can cause damage to the sensory nerves, resulting in hypoesthesia and diminished temperature sensation, leading to insensitivity to external stimuli and skin fragility [37]. When the motor nerve is damaged, toe deformity and changes in gait can occur, resulting in abnormal local and plantar pressure loads that cause calluses and ulcers [38]. In addition, DPN-induced impairment of sweat nerve function can cause atrophy of sweat glands, a weakened perspiration response, and dry skin, resulting in pruritus, chapped skin, and foot skin ulcers [39]. According to a study of 142 people with diabetes, impaired sweat secretion may eventually weaken the skin barrier and increase the risk of foot ulcers [40]. DPN patients with abnormal sweating function have a 15-fold increased risk of foot ulcers [41]. The visual indicator plaster method has a high sensitivity and low negative predictive value for the diagnosis of DPN and is simple to perform for the assessment of foot skin dryness [42]. The Sudoscan technology evaluates the degree of neurological impairment of sweat glands by detecting changes in chloride ion concentration in the palm and sole sweat to provide a quantitative analysis of sweating function [43].

Most diabetic patients exhibit oral symptoms, including periodontal disease, tooth loss, dental caries, dry mouth, delayed wound healing, and taste dysfunction [44]. It is widely recognized that periodontitis and diabetes interact in both directions [45]. Periodontitis is a chronic, long-lasting inflammation frequently accompanied by gum infection, periodontal redness, bleeding gums, loose teeth, tooth sensitivity, and persistent bad breath [46]. Periodontitis causes immune impairment and systemic inflammation, which can result in dysregulation of bacterial flora, decreased function of islet cells, insulin resistance, impaired fasting blood glucose, and endothelial dysfunction, thus interfering with normal glucose metabolism [[47], [48], [49], [50]]. In patients with diabetes mellitus, the prevalence of severe periodontitis increases three-to fourfold, and the severity is associated with inadequate glycemic control [51]. Numerous epidemiological studies, clinical intervention studies, and animal experiments have supported the hypothesis that periodontitis may be an independent risk factor for diabetes mellitus, resulting in a poor diabetes prognosis [[52], [53], [54]]. In the interim, the incidence of diabetes in patients with severe periodontitis increased by 53% [52]. In generally healthy individuals, periodontitis can significantly increase the risk of developing diabetes, with adverse effects on glycemic control, diabetic complications, and the development of TD2M [55]. Periodontal disease and increased missing teeth may be risk factors for developing new-onset diabetes [56]. In adults with diabetes mellitus, the likelihood of tooth loss was double that of adults without tooth loss [57]. Therefore, adults with diabetes mellitus must be encouraged to maintain good oral hygiene and teeth [58].

According to the color, texture, shape, and fur characteristics of the panoramic tongue image, a proposed TCM-based diabetes diagnosis method has good classification performance in the early stage of the disease. Therefore, it can be used as a preliminary screening procedure for the future early detection of T2DM and its complications [59]. Doctors have traditionally examined these tongue characteristics based on years of subjective experience to aid treatment selection and prognosis [60,61]. Studies have correlated a yellow tongue coating with an increased risk of diabetes [62,63]. Through traditional machine learning and deep learning algorithms, a standardized, objectified, and quantified tongue image diagnosis method and an automatic tongue diagnosis system have been established and developed, with improved prediction accuracy and the ability to increase the consistency of subjective diabetes diagnosis [22,64,65]. Some TCM indexes (tongue features and symptoms), such as chest tightness, spontaneous perspiration, a wiry pulse, and greasy tongue coating, can also improve the diagnosis of metabolic syndrome, according to Xia et al. who compared three classic machine learning methods [66]. Japanese non-smoking men and women with a yellow tongue coating had a higher prevalence of diabetes mellitus, according to a previous epidemiological study [62]. Blood stasis is regarded as one of the primary accompanying syndromes of DPN in TCM. Li et al. discovered that 89–96% of patients with DPN have blood stasis [67]. Moreover, Morita A. et al. Demonstrated an objective evaluation of the relationship between blood stasis and patients with a dark purple tongue [65]. The performance of the tongue is essential for detecting blood stasis. A study indicated that patients with T2DM have increased blood stasis tongue manifestations, which are correlated with severe arterial stiffness [68]. Meanwhile, this is the first study to identify the purple tongue as an independent risk factor positively associated with DPN.

### 4.1 Limitations

First, the populations were recruited from a single center, so whether these results apply to other regions is still being determined. Because tongue characteristics vary regionally in DPN, we will evaluate some of these indicators in future research to supplement the existing models. Second, we constructed our model based on demographic characteristics. TCM symptoms and tongue features were assessed using an anthropometric questionnaire, but other variables such as biochemical parameters were not included in the evaluation. Third, despite the lack of evidence, other TCM symptoms and tongue characteristics don't include as variables in our study may be associated with the occurrence and development of DPN risk in T2DM.

## 5 Conclusion

The eight variables about DPN confirmed in his analysis, including age, smoking, insomnia, sweating, loose teeth, dry skin, purple tongue, and dark red tongue, have great clinical application value for identifying DPN risk in T2DM, particularly for TCM clinical doctors. In addition, it can assist clinical personnel in providing a reference for screening and early diagnosis of DPN by incorporating TCM patient symptoms and tongue characteristics into the risk prediction model and calculating the risk score to assess the risk of DPN in T2DM patients.

## Author contributions

1) conceived and designed the experiments by ZT, YF and HW; 2) performed the experiments by ZT, XS, DW, JG, YG, YZ, HB and ZZ; 3) analyzed and interpreted the data by ZT, XS, and ZW; 4) contributed reagents, materials, analysis tools or data by ZT, YF, HW and ZW; 5) wrote the paper by ZT, YF, HW.

## Funding

This project was supported by the 10.13039/501100012166National Key Research and Development Program of China (approval No.: 2017YFC1703305) and Hebei Administration of Traditional Chinese Medicine 2022 Scientific Research Program of Traditional Chinese Medicine (approval No.: 2022554) during the 13th 5-year plan period.

## Data availability statement

Data will be made available on request.

## Declaration of competing interest

The authors declare that they have no known competing financial interests or personal relationships that could have appeared to influence the work reported in this paper.

**Table S1:** 

Clinical characteristics N (%)	T2DM (4430)	DPN (293)	P value
Age			0.000
18～44	256 (5.8)	9 (3.1)	
45～59	1280 (28.9)	66 (22.5)	
60～89	2687 (60.7)	185 (63.1)	
>90	207 (4.7)	33 (11.3)	
BMI			0.023
<18.5	39 (0.9)	6 (2)	
18.5～24.5	1806 (40.8)	110 (37.5)	
25～28	1990 (44.9)	124 (42.3)	
>28	539 (13.4)	53 (18.1)	
sex			0.000
male	2028 (45.8)	168 (57.3)	
female	2402 (54.2)	125 (42.7)	
smoke	971 (21.9)	130 (44.4)	0.000
fatigue	2594 (58.6)	169 (57.7)	0.768
irritable	1739 (39.3)	113 (38.6)	0.815
forgetfulness	181 (4.1)	9 (3.1)	0.392
insomnia	2061 (46.5)	108 (36.9)	0.001
sweating	1199 (27.1)	45 (15.4)	0.000
loose teeth	1127 (25.4)	108 (36.9)	0.000
dry skin	717 (16.2)	86 (29.4)	0.000
dry mouth	1820 (41.1)	120 (41)	0.966
polydipsia	701 (15.8)	54 (18.4)	0.238
thirst does not drink much	114 (2.6)	12 (4.1)	0.117
polyuria	162 (3.7)	20 (6.8)	0.006
frequency of urine	562 (12.7)	34 (11.6)	0.589
crimson tongue	700 (15.8)	55 (18.8)	0.179
purple tongue	329 (7.4)	44 (15)	0.000
Dark red tongue	513 (11.6)	3 (1)	0.000
enlarged tongue	449 (10.1)	21 (7.2)	0.100
spotted tongue	285 (6.4)	21 (7.2)	0.621
teeth marked tongue	1119 (25.3)	62 (21.2)	0.117
fissured tongue	1658 (37.4)	101 (34.5)	0.311
yellow coating	1162 (26.2)	103 (35.2)	0.001
less coating	305 (6.8)	23 (7.8)	0.529
thick coating	1040 (23.5)	55 (18.8)	0.065
greasy coating	1149 (25.9)	51 (17.4)	0.001

## References

[1] International Diabetes Federation (2021).

[2] Wang L., Peng W., Zhao Z., Zhang M., Shi Z., Song Z. (2021). Prevalence and treatment of diabetes in China, 2013-2018. JAMA.

[3] Tu W.-J., Xue Y., Nie D. (2022). The prevalence and treatment of diabetes in China from 2013 to 2018. JAMA.

[4] Sloan G., Selvarajah D., Tesfaye S. (2021). Pathogenesis, diagnosis and clinical management of diabetic sensorimotor peripheral neuropathy. Nat. Rev. Endocrinol..

[5] Paisey R.B., Abbott A., Levenson R., Harrington A., Browne D., Moore J. (2018). Diabetes-related major lower limb amputation incidence is strongly related to diabetic foot service provision and improves with enhancement of services: peer review of the South-West of England. Diabet. Med..

[6] Zhang Y., Lazzarini P.A., McPhail S.M., van Netten J.J., Armstrong D.G., Pacella R.E. (2020). Global disability burdens of diabetes-related lower-extremity complications in 1990 and 2016. Diabetes Care.

[7] Mizokami-Stout K.R., Li Z., Foster N.C., Shah V., Aleppo G., McGill J.B. (2020). The contemporary prevalence of diabetic neuropathy in type 1 diabetes: findings from the T1D exchange. Diabetes Care.

[8] Jeyam A., McGurnaghan S.J., Blackbourn L.A.K., McKnight J.M., Green F., Collier A. (2020). Diabetic neuropathy is a substantial burden in people with type 1 diabetes and is strongly associated with socioeconomic disadvantage: a population-representative study from scotland. Diabetes Care.

[9] Braffett B.H., Gubitosi-Klug R.A., Albers J.W., Feldman E.L., Martin C.L., White N.H. (2020). Risk factors for diabetic peripheral neuropathy and cardiovascular autonomic neuropathy in the diabetes control and complications trial/epidemiology of diabetes interventions and complications (DCCT/EDIC) study. Diabetes.

[10] Feldman E.L., Callaghan B.C., Pop-Busui R., Zochodne D.W., Wright D.E., Bennett D.L. (2019). Diabetic neuropathy. Nat. Rev. Dis. Prim..

[11] Callaghan B.C., Little A.A., Feldman E.L., Hughes R.A.C. (2012). Enhanced glucose control for preventing and treating diabetic neuropathy. Cochrane Database Syst. Rev..

[12] Haque F., Ibne Reaz M.B., Chowdhury M.E.H., Md Ali S.H., Ashrif A., Bakar A., Rahman T. (2021). A nomogram-based diabetic sensorimotor polyneuropathy severity prediction using Michigan neuropathy screening instrumentations. Comput. Biol. Med..

[13] Tesfaye S., Boulton A.J.M., Dyck P.J., Freeman R., Horowitz M., Kempler P. (2010). Diabetic neuropathies: update on definitions, diagnostic criteria, estimation of severity, and treatments. Diabetes Care.

[14] England J.D., Gronseth G.S., Franklin G., Miller R.G., Asbury A.K., Carter G.T. (2005). Distal symmetric polyneuropathy: a definition for clinical research: report of the American academy of neurology, the American association of electrodiagnostic medicine, and the American academy of physical medicine and rehabilitation. Neurology.

[15] Pop-Busui R., Boulton A.J.M., Feldman E.L., Bril V., Freeman R., Malik R.A. (2017). Diabetic neuropathy: a position statement by the American diabetes association. Diabetes Care.

[16] Devigili G., Rinaldo S., Lombardi R., Cazzato D., Marchi M., Salvi E. (2019). Diagnostic criteria for small fibre neuropathy in clinical practice and research. Brain.

[17] Sloan G., Selvarajah D., Tesfaye S. (2021). Pathogenesis, diagnosis and clinical management of diabetic sensorimotor peripheral neuropathy. Nat. Rev. Endocrinol..

[18] Selvarajah D., Kar D., Khunti K., Davies M.J., Scott A.R., Walker J. (2019). Diabetic peripheral neuropathy: advances in diagnosis and strategies for screening and early intervention. Lancet Diabetes Endocrinol..

[19] Chinese Diabetes Association (2018). Chinese guidelines for the prevention and treatment of type 2 diabetes (2017 edition). Zhonghua Tang Niao Bing Za Zhi.

[20] Chen C., Lu F.C., Department of Disease Control Ministry of Health, PR China (2004). The guidelines for prevention and control of overweight and obesity in Chinese adults. Biomed. Environ. Sci..

[21] Jian-feng Z., Jia-tuo X., Li-ping T., Zhi-feng Z., Xiao-juan H., Ji C. (2017). Study on the characteristics of sub-health symptoms and TCM syndrome patterns distribution in 1754 non-disease population. Chin. J. Integr. Med..

[22] Zhang J., Xu J., Hu X., Chen Q., Tu L., Huang J. (2017). Diagnostic method of diabetes based on support vector machine and tongue images. BioMed Res. Int..

[23] Friedman J., Hastie T., Tibshirani R. (2010). Regularization paths for generalized linear models via coordinate descent. J. Stat. Software.

[24] Li L., Wang L., Zeng F., Peng G., Ke Z., Liu H. (2021). Development and multicenter validation of a CT-based radiomics signature for predicting severe COVID-19 pneumonia. Eur. Radiol..

[25] Harrell F.E., Califf R.M., Pryor D.B., Lee K.L., Rosati R.A. (1982). Evaluating the yield of medical tests. JAMA.

[26] Wu S., Zhang X., Rui W., Sheng Y., Yu Y., Zhang Y. (2022). A nomogram strategy for identifying the subclassification of IDH mutation and ATRX expression loss in lower-grade gliomas. Eur. Radiol..

[27] Collins G.S., Reitsma J.B., Altman D.G., Moons K.G.M. (2015). Transparent reporting of a multivariable prediction model for individual prognosis or diagnosis (TRIPOD): the TRIPOD statement. J. Br. Surg..

[28] Yan P., Zhang Z., Miao Y., Xu Y., Zhu J., Wan Q. (2019). Physiological serum total bilirubin concentrations were inversely associated with diabetic peripheral neuropathy in Chinese patients with type 2 diabetes: a cross-sectional study. Diabetol. Metab. Syndrome.

[29] Lu B., Yang Z., Wang M., Yang Z., Gong W., Yang Y. (2010). High prevalence of diabetic neuropathy in population-based patients diagnosed with type 2 diabetes in the Shanghai downtown. Diabetes Res. Clin. Pract..

[30] Papanas Nikolaos, Ziegler Dan (2015). Risk factors and comorbidities in diabetic neuropathy: an update 2015. Rev. Diabet. Stud.: Reg. Dev. Stud..

[31] Darivemula S., Nagoor K., Patan S.K., Reddy N.B., Deepthi C.S., Chittooru C.S. (2019). Prevalence and its associated determinants of diabetic peripheral neuropathy (DPN) in individuals having type-2 diabetes mellitus in rural south India. Indian J. Community Med..

[32] Li L., Chen J., Wang J., Cai D. (2015). Prevalence and risk factors of diabetic peripheral neuropathy in Type 2 diabetes mellitus patients with overweight/obese in Guangdong province, China. Prim. Care Diabetes.

[33] Jaiswal M., Divers J., Dabelea D., Isom S., Bell R.A., Martin C.L. (2017). Prevalence of and risk factors for diabetic peripheral neuropathy in youth with type 1 and type 2 diabetes: SEARCH for diabetes in youth study. Diabetes Care.

[34] Liu X., Bragg F., Yang L., Kartsonaki C., Guo Y., Du H. (2018). Smoking and smoking cessation in relation to risk of diabetes in Chinese men and women: a 9-year prospective study of 0· 5 million people. Lancet Public Health.

[35] Pan A., Wang Y., Talaei M., Hu F.B., Wu T. (2015). Relation of active, passive, and quitting smoking with incident type 2 diabetes: a systematic review and meta-analysis. Lancet Diabetes Endocrinol..

[36] Zhang P., Lu J., Jing Y., Tang S., Zhu D., Bi Y. (2017). Global epidemiology of diabetic foot ulceration: a systematic review and meta-analysis. Ann. Med..

[37] AlAyed M.Y., Younes N., Al-Smady M., Khader Y.S., Robert A.A., Ajlouni K. (2017). Prevalence of foot ulcers, foot at risk and associated risk factors among Jordanian diabetics. Curr. Diabetes Rev..

[38] Yazdanpanah L., Shahbazian H., Nazari I., Hesam S., Ahmadi F., Cheraghian B. (2018). Risk factors associated with diabetic foot ulcer-free survival in patients with diabetes. Diabet.Diabet. Metab. Res. Rev. Metabol. Syndr..

[39] Volmer-Thole M., Lobmann R. (2016). Neuropathy and diabetic foot syndrome. Int. J. Mol. Sci..

[40] Gin H., Baudoin R., Raffaitin C.H., Rigalleau V., Gonzalez C. (2011). Non-invasive and quantitative assessment of sudomotor function for peripheral diabetic neuropathy evaluation. Diabetes Metab..

[41] Tentolouris N., Marinou K., Kokotis P., Karanti A., Diakoumopoulou E., Katsilambros N. (2009). Sudomotor dysfunction is associated with foot ulceration in diabetes. Diabet. Med..

[42] Ziegler D., Papanas N., Rathmann W., Heier M., Scheer M., Meisinger C., KORA Study Group (2012). Evaluation of the Neuropad sudomotor function test as a screening tool for polyneuropathy in the elderly population with diabetes and pre-diabetes: the KORA F4 survey. Diabetes Metab Res Rev.

[43] Burgess J., Frank B., Marshall A., Khalil R.S., Ponirakis G., Petropoulos I.N. (2021). Early detection of diabetic peripheral neuropathy: a focus on small nerve fibres. Diagnostics.

[44] Nazir M.A., AlGhamdi L., AlKadi M., AlBeajan N., AlRashoudi L., AlHussan M. (2018). The burden of diabetes, its oral complications and their prevention and management. Open Access Maced J. Med. Sci..

[45] Preshaw P.M., Alba A.L., Herrera D., Jepsen S., Konstantinidis A., Makrilakis K. (2012). Periodontitis and diabetes: a two-way relationship. Diabetologia.

[46] Chapple I.L.C., Genco R. (2013). Working group 2 of the joint EFP/AAP workshop. Diabetes and periodontal diseases: consensus report of the Joint EFP/AAP Workshop on Periodontitis and Systemic Diseases. J. Periodontol..

[47] Preshaw P.M., Bissett S.M. (2019). Periodontitis and diabetes. Br. Dent. J..

[48] Islam S.K., Seo M., Lee Y.S., Moon S.S. (2015). Association of periodontitis with insulin resistance, β-cell function, and impaired fasting glucose before onset of diabetes. Endocr. J..

[49] Blasco-Baque V., Garidou L., Pomié C., Escoula Q., Loubieres P., Le Gall-David (2017). Periodontitis induced by Porphyromonas gingivalis drives periodontal microbiota dysbiosis and insulin resistance via an impaired adaptive immune response. Gut.

[50] Tian J., Liu C., Zheng X., Jia X., Peng X., Yang R. (2020). Porphyromonas gingivalis induces insulin resistance by increasing BCAA levels in mice. J. Dent. Res..

[51] Lalla E., Papapanou P.N. (2011). Diabetes mellitus and periodontitis: a tale of two common interrelated diseases. Nat. Rev. Endocrinol..

[52] Wu C.Z., Yuan Y.H., Liu H.H., Li S.S., Zhang B.W., Chen W. (2020). Epidemiologic relationship between periodontitis and type 2 diabetes mellitus. BMC Oral Health.

[53] Abariga Samuel A., Whitcomb Brian W. (2016). Periodontitis and gestational diabetes mellitus: a systematic review and meta-analysis of observational studies. BMC Pregnancy Childbirth.

[54] Demmer R.T., Jacobs D.R., Desvarieux M. (2008). Periodontal disease and incident type 2 diabetes: results from the First National Health and Nutrition Examination Survey and its epidemiologic follow-up study. Diabetes Care.

[55] Baeza M., Morales A., Cisterna C., Cavalla F., Jara G., Isamitt Y. (2020). Effect of periodontal treatment in patients with periodontitis and diabetes: systematic review and meta-analysis. J. Appl. Oral Sci..

[56] Chang Y., Lee J.S., Lee K.-J., Woo H.G., Song T.-J. (2020). Improved oral hygiene is associated with decreased risk of new-onset diabetes: a nationwide population-based cohort study. Diabetologia.

[57] AlDukhail S., Alhazmi H., Riedy C., Barrow J.R., Chamut S. (2021). Oral health outcomes among adults with diabetes served at HRSA-funded health centers. J. Diabet. Complicat..

[58] Luo H., Pan W., Sloan F., Feinglos M., Wu B. (2015). Forty-year trends in tooth loss among American adults with and without diabetes mellitus: an age-period-cohort analysis. Prev. Chronic Dis..

[59] Zhang Bob, Vijaya Kumar B.V.K., Zhang David (2013). Detecting diabetes mellitus and nonproliferative diabetic retinopathy using tongue color, texture, and geometry features. IEEE Trans. Biomed. Eng..

[60] Jung C.J., Jeon Y.J., Kim J.Y., Kim K.H. (2012). Review on the current trends in tongue diagnosis systems. Integr. Med. Res..

[61] Thirunavukkarasu U., Umapathy S., Krishnan P.T., Janardanan K. (2020). Human tongue thermography could Be a prognostic tool for prescreening the type II diabetes mellitus. Evid. Base. Compl. Alternative Med..

[62] Tomooka K., Saito I., Furukawa S., Maruyama K., Eguchi E., Iso H. (2018). Yellow tongue coating is associated with diabetes mellitus among Japanese non-smoking men and women: the toon health study. J. Epidemiol..

[63] Hsu P.-C., Wu H.-K., Huang Y.-C., Chang H.-H., Lee T.-C., Chen Y.-P. (2019). The tongue features associated with type 2 diabetes mellitus. Medicine (Baltim.).

[64] Li J., Yuan P., Hu X., Huang J., Cui L., Cui J. (2021). A tongue features fusion approach to predicting prediabetes and diabetes with machine learning. J. Biomed. Inf..

[65] Morita A., Murakami A., Noguchi K., Watanabe Y., Nakaguchi T., Ochi S. (2021). Combination image analysis of tongue color and sublingual vein improves the diagnostic accuracy of oketsu (blood stasis) in kampo medicine. Front. Med..

[66] Xia S.-J., Gao B.-Z., Wang S.-H., Guttery D.S., Li C.-D., Zhang Y.-D. (2021). Modeling of diagnosis for metabolic syndrome by integrating symptoms into physiochemical indexes. Biomed. Pharmacother..

[67] Li M., Su H., Xiang Y., Zhang J., Zhao J., Lin L. (2013). Features analysis on Traditional Chinese Medicine syndromes in patients with diabetic peripheral neuropathy. J. Tradit. Chin. Med..

[68] Hsu P.C., Huang Y.C., Chiang J.Y., Chang H.H., Liao P.Y., Lo L.C. (2016). The association between arterial stiffness and tongue manifestations of blood stasis in patients with type 2 diabetes. BMC Compl. Alternative Med..

